# Milk and Tuberculosis

**Published:** 1966-01

**Authors:** A. D. Osborne, A. V. Neale

**Affiliations:** Senior Lecturer, Veterinary Public Health; Emeritus Professor of Child Health, University of Bristol


### MILK AND TUBERCULOSIS

BY

A. D. OSBORNE

*Senior Lecturer, Veterinary Public Health,*

AND

A. V. NEALE

*Emeritus Professor of Child Health, University of Bristol.*

Following the first isolation of bovine tubercle bacilli in a child in 1902, the significance of the cattle strain in human disease rapidly became obvious. (Table 1).

TABLE I

Mortality per million living at all ages. 1902 Registrar General's Report, England and Wales

| | Age – years | | | |
|---|---|---|---|---|
| | 0– | 5– | 10– | 15– |
| Tuberculous meningitis | 3188 | 801 | 358 | 198 |
| Tuberculous peritonitis | 2790 | 316 | 212 | 172 |

The work of Branson (1905) gave some indication of the incidence of abdominal tuberculosis in children which was for the most part the result of infection by this strain.

At this time it was known that there was a high incidence of tuberculosis in milking cows. For instance, 46·8 per cent of cows slaughtered in London in 1901 showed lesions (King 1901) a figure which dropped to 33 per cent for the years 1918–27. (Young 1929). Even in the early 1940's, however, figures from various slaughterhouses showed an incidence of over 40 per cent in milking cows (Francis 1947).

During the period from 1930 to 1963 the incidence of bovine tuberculosis infection in children and adults, as indicated by the incidence of death from tuberculosis of the intestines and of joints, showed a reduction (see Table 2).

TABLE 2

Deaths – all ages. England and Wales. Registrar General's Reports.

Actual number of Deaths

| Year | Tuberculosis: all forms | Tuberculosis: Intestines and Peritoneum | Tuberculosis: Bones and Joints |
|---|---|---|---|
| 1930 | 35,745 | 1,149 | 691 |
| 1945 | 23,464 | 546 | 476 |
| 1950 | 13,806 | 182 | 355 |
| 1955 | 6,492 | 86 | 198 |
| 1959 | 3,854 | 38 | 66 |
| 1963 | 2,960 | 39 | 45 |

In 1950 a compulsory scheme for the eradication of bovine tuberculosis was put into operation and in the short period of ten years the incidence of tuberculous cows slaughtered at one large abattoir fell from 37·71 per cent to 3·22 per cent. This has now been further reduced to 0·31 per cent (Hughes 1964) and the incidence of reactors to the tuberculin test in Great Britain had dropped to 0·11 per cent of all cattle in 1962 (Report 1964).

It is interesting to note that paradoxically there seems now to be an almost greater chance that the cow is infected from the human as was demonstrated by Hargreaves (1961) when an "epidemic" of reactors followed the employment of a tuberculous farm worker.

It would be tempting to make a direct correlation between these two trends of the last 15 years but it must be remembered that the period has also seen a noticeable increase in the extent of pasteurization. The figure for 1949–50 was 79·1 per cent of total liquid milk sales whilst that for 1963–64 was 94·7 per cent (Smith 1965). The 5·3 per cent of unpasteurized milk (designated "untreated" for sale to the general public) is mainly sold by producer retailers in rural or semirural areas. Although such milk could be a source of a tuberculous infection, the chances seem to be relatively remote when it is realized that only one in a hundred of tuberculous cows were found to be affected in the udder, and the present incidence of bovine infection is certainly less than one in a thousand. At most, therefore, there is a possibility of only one in 100,000 cows excreting the organism, i.e. perhaps 30 cows in the whole country. There is obviously a much greater risk of infection by *Br. abortus*, for the latest survey (M.A.F.F. 1965) shows that an average of 2·1 per cent of the national herd is excreting this organism in the milk. It is of course fortunate that the main justification for pasteurization has now become its effect on the keeping quality of the milk, for this will ensure a continuation of the process while risks, however small, of milk infection remain. It is to be hoped, however, that while the T.B. eradication programme is being carried to its ultimate successful conclusion the Treasury will make funds available for the initiation of a similar scheme to control and eradicate brucellosis as has been suggested by the B.V.A. (1965).

The combined result of almost complete eradication of bovine tuberculosis and pasteurization has been to reduce to an almost insignificant level the chances of childhood infection by what was once a serious and common cause of ill health and, not infrequently, of death. Sir Robert Phillip's (1937) urgent plea for a "pure, sound, tubercle-free milk produced on a commercial basis and tubercle-free herds everywhere" has become an actual achievement. But it is maintained only by expert and continued vigilance. In 1963 there were almost 3 million cows in England, and Scotland and Wales, and over 2,000 million gallons of T.T. milk were distributed. About fifty million gallons were supplied as "school milk" and two hundred million gallons as "welfare milk". A great improvement in the quality of this vast quantity of cows' milk has already been achieved. Further improvement is possible and measures have already been suggested. Such facts are obviously of tremendous importance to the National Health!

### REFERENCES

Branson, W. P. S. (1905). "Abdominal Tuberculosis in Childhood". *Med. Chir. trans. (Lond.)* **88**, 349.

British Veterinary Association (1965). *Vet. Rec.* **77**, 135.

Francis, J. (1947). *Bovine Tuberculosis.* Staples Press Ltd., London.

Hargreaves, E. R. (1961). "Epidemiological studies in Cornwall: Tuberculosis", *Proc. Roy. Soc. Med.* **54**, 214.

Hughes, J. M. (1964). Personal communication.
King, J. (1901). "Tuberculosis and the Meat Supply", *Lancet* **2**, 245.
Ministry of Agriculture, Fisheries and Food (1964). Animal Diseases Survey, Report No. [illegible] Brucellosis in the British Dairy Herd, London, H.M.S.O.
Phillip, R. (1937). Collected Papers on Tuberculosis. London.
Registrar General's Statistical Review: England and Wales. Reports. 1901–1963.
Report (1964). Report on the Animal Health Services in Great Britain 1961 and 19[illegible] London, H.M.S.O.
Smith, G. F. (1965). Personal communication.
Young, T. D. (1929), cited by Savage, W. G. The prevention of human tuberculosis of bovi[illegible] origin, pp. 195. London.

